# Behavioral responses to predatory sounds predict sensitivity of cetaceans to anthropogenic noise within a soundscape of fear

**DOI:** 10.1073/pnas.2114932119

**Published:** 2022-03-21

**Authors:** Patrick J. O. Miller, Saana Isojunno, Eilidh Siegal, Frans-Peter A. Lam, Petter H. Kvadsheim, Charlotte Curé

**Affiliations:** ^a^Sea Mammal Research Unit, University of St Andrews, St Andrews, Fife KY16 9QQ, United Kingdom;; ^b^Acoustics & Sonar, Netherlands Organisation for Applied Scientific Research, NL-2509 The Hague, The Netherlands;; ^c^Sensor and Surveillance Systems, Norwegian Defence Research Establishment, 2007 Horten, Norway;; ^d^Cerema, University Gustave Eiffel, UMRAE, F-67210 Strasbourg, France

**Keywords:** evolution, Cetacea, disturbance, naval sonar, risk–disturbance hypothesis

## Abstract

As human activities impact virtually every animal habitat on the planet, identifying species at-risk from disturbance is a priority. Cetaceans are an example taxon where responsiveness to anthropogenic noise can be severe but highly species and context specific, with source–receiver characteristics such as hearing sensitivity only partially explaining this variability. Here, we predicted that ecoevolutionary factors that increase species responsiveness to predation risk also increase responsiveness to anthropogenic noise. We found that reductions in intense-foraging time during exposure to 1- to 4-kHz naval sonar and predatory killer whale sounds were highly correlated (*r* = 0.92) across four cetacean species. Northern bottlenose whales ceased foraging completely during killer whale and sonar exposures, followed by humpback, long-finned pilot, and sperm whales, which reduced intense foraging by 48 to 97%. Individual responses to sonar were partly predicted by species-level responses to killer whale playbacks, implying a similar level of perceived risk. The correlation cannot be solely explained by hearing sensitivity, indicating that species- and context-specific antipredator adaptations also shape cetacean responses to human-made noise. Species that are more responsive to predator presence are predicted to be more disturbance sensitive, implying a looming double whammy for Arctic cetaceans facing increased anthropogenic and predator activity with reduced ice cover.

## Significance

Acoustic signals travel efficiently in the marine environment, allowing soniferous predators and prey to eavesdrop on each other. Our results with four cetacean species indicate that they use acoustic information to assess predation risk and have evolved mechanisms to reduce predation risk by ceasing foraging. Species that more readily gave up foraging in response to predatory sounds of killer whales also decreased foraging more during 1- to 4-kHz sonar exposures, indicating that species exhibiting costly antipredator responses also have stronger behavioral reactions to anthropogenic noise. This advance in our understanding of the drivers of disturbance helps us to predict what species and habitats are likely to be most severely impacted by underwater noise pollution in oceans undergoing increasing anthropogenic activities.

Why are some species more averse to anthropogenic noise disturbances than others? Comparative frameworks for species sensitivity are urgently needed as human activities impact the marine environment on a global scale (1), with underwater noise from shipping, seismic exploration, and military sonar (2) and increased activities in the Arctic Ocean being of particular concern (3). Auditory sensitivity and acoustic masking have been the dominant explanatory factors when comparing the sensitivity of marine organisms that rely on sound for critical life functions (4, 5). However, both theoretical and empirical work have shown that evolutionary and ecological context variables (hereafter, ecoevolutionary factors) other than hearing sensitivity, such as antipredator adaptations and habitat quality, can also be expected to play a significant role (4, 6–8). This can be illustrated in cetaceans that use underwater sound as a primary sensory and communication modality and for which research efforts have characterized and quantified a diverse array of noise-induced behavioral effects (5, 9–11). Experimental sound exposures show that free-ranging cetaceans respond to noise by ceasing fitness-enhancing activities, such as feeding (12), leading to concern over population-level impacts (13). Responsiveness varies across species (14), with some taxa like beaked whales (14–16) and harbor porpoises (17, 18) considered to be particularly sensitive. Nevertheless, it remains unclear to which extent antipredator adaptations versus other ecoevolutionary factors, like auditory sensitivity (5), might drive this variation (4, 10). Crucially, to our knowledge, predictions linking antipredator adaptations and noise disturbance have not been quantitatively tested in a unified analysis across different species sharing the same underwater soundscape.

The risk–disturbance hypothesis posits that responses to a disturbance source are the outcome of each animals’ internal trade-off between the perceived risk posed, against the fitness and missed opportunity costs of a response (6). Given that antipredator responses are costly, prey are expected to adjust their response thresholds according to the phenotypic and evolutionary contexts that have shaped their responses to predation risk (19–22). We thus predicted that species and/or populations that are in ecoevolutionary contexts more vulnerable to predation should respond more strongly to both predation risk and anthropogenic disturbances. On the other hand, contexts that promote tolerance of predation risk, such as higher-risk/higher-reward foraging, are expected to also translate to tolerance of anthropogenic disturbance.

We empirically tested this prediction by comparing changes in foraging time budgets of four cetacean species (northern bottlenose, humpback, sperm, and long-finned pilot whales) in their feeding grounds during experimental exposure to 1- to 4-kHz naval sonar and predatory mammal-eating killer whale sound (hereafter KW-mammal) playbacks. Playbacks of killer whale sounds elicit antipredator behavior in seals (23) and cetaceans (24–26), providing a yardstick for costly and aversive reactions that have evolved to reduce predation risk (27). We chose to quantify reductions in foraging time budgets because that is a well-defined and quantifiable behavioral change that reflects the trade-off between food and safety, which is shared across animal taxa (28). Most mesopredator cetaceans are known prey of killer whales (29), although the precise extent to which each species is subject to predation remains poorly understood. Diverse antipredator strategies across the four species in our study imply a priori that variation in the strength of antipredator responses is expected. Large adult male sperm whales in our study and adult humpback whales with long flippers have strong fight capabilities (30), while large groups of long-finned pilot whales may use social mobbing responses against predation threats (25). In contrast, the northern bottlenose whale, with no physical defense and smaller group sizes, likely relies upon crypsis and flight to avoid predation as do other beaked whales (31). Echolocation sounds of toothed whales while foraging (32) and body movements of lunge-feeding baleen whales (33) are conspicuous, exposing foragers to increased predation risk (e.g., reference 34); consequently, cryptic antipredator responses imply cessation of feeding that will carry a clear consequence to energetic balance. Furthermore, predator detection by prey may be less effective during foraging (28). Therefore, foraging time represents a quantifiable and sensitive indicator of responsiveness to a threat, which can be applied to the diverse species in our study.

**Fig. 1.**
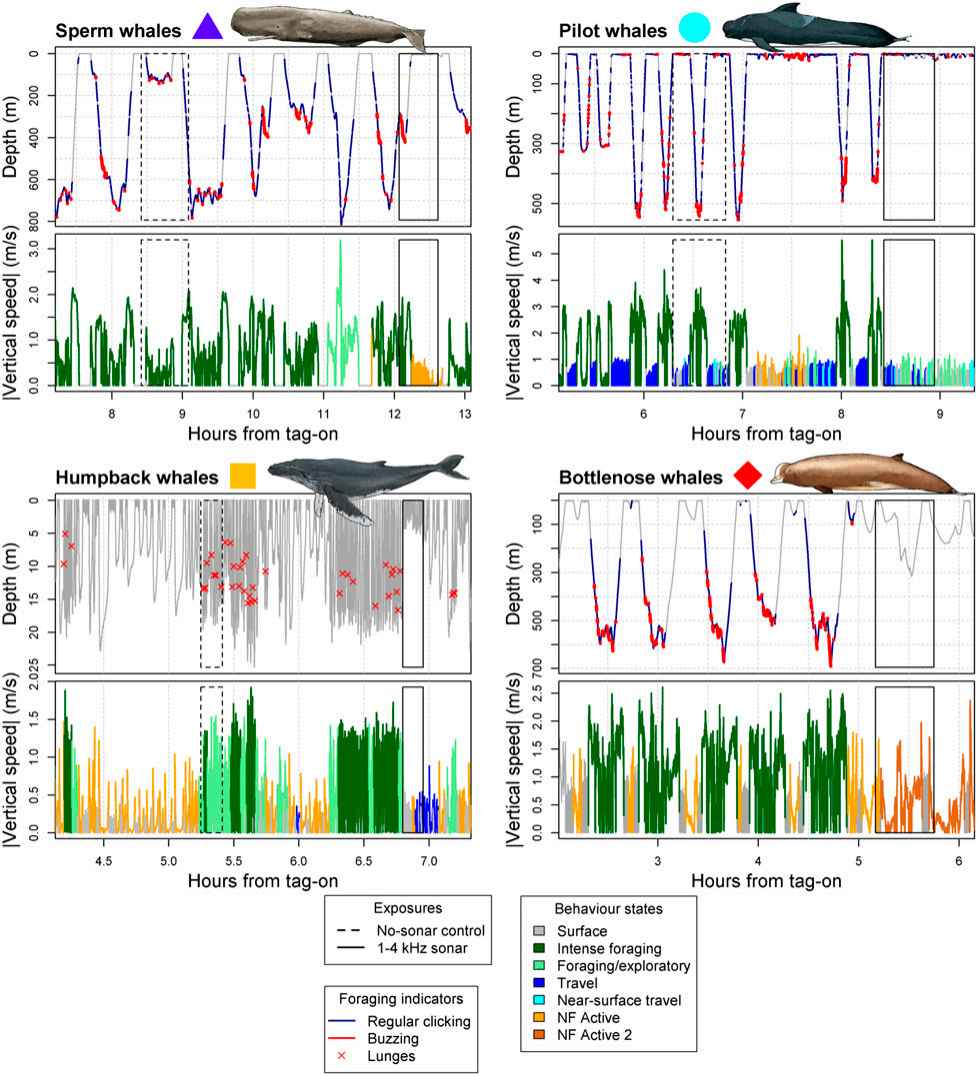
Representative time series behavioral data recorded by sound-and-movement recording Dtags, with exposure periods marked as boxes. For each species, the *Top* panels show dive depth versus time, with feeding indicators shown in color (navy blue, echolocation click production; red line, buzz clicks; red crosses, lunges). *Bottom* panels show the absolute value of vertical speed, with the color indicating the behavioral state. Note the dark-green intense foraging state was associated with feeding indicators and higher vertical speeds. Note the reduction in intense foraging during 1- to 4-kHz sonar treatments (solid boxes) but little effect of the no-sonar control treatment (dashed boxes).

Sound and movement data from suction-cup-attached data loggers were used to classify dives of 43 whales of 4 species (*SI Appendix*, Table S1) into functional states, including intense foraging—when animals were maximally engaged in foraging related echolocation or movement behaviors (Fig. 1 and *SI Appendix*, Table S2). We quantified how time spent in intense foraging during baseline periods changed during exposures to 1- to 4-kHz sonar and predator sounds (KW-mammal). We expected a priori that 1) both stimuli elicit a reduction in intense foraging time, 2) responses to predator sounds are stronger than to 1- to 4-kHz sonar, and 3) higher species average responses to predator sounds correspond with higher responsiveness to 1- to 4-kHz sonar because the ecoevolutionary drivers that increase responsiveness to predation risk are also expected to increase responsiveness to anthropogenic threats in each species' study population and environmental context.

## Results

**Fig. 2.**
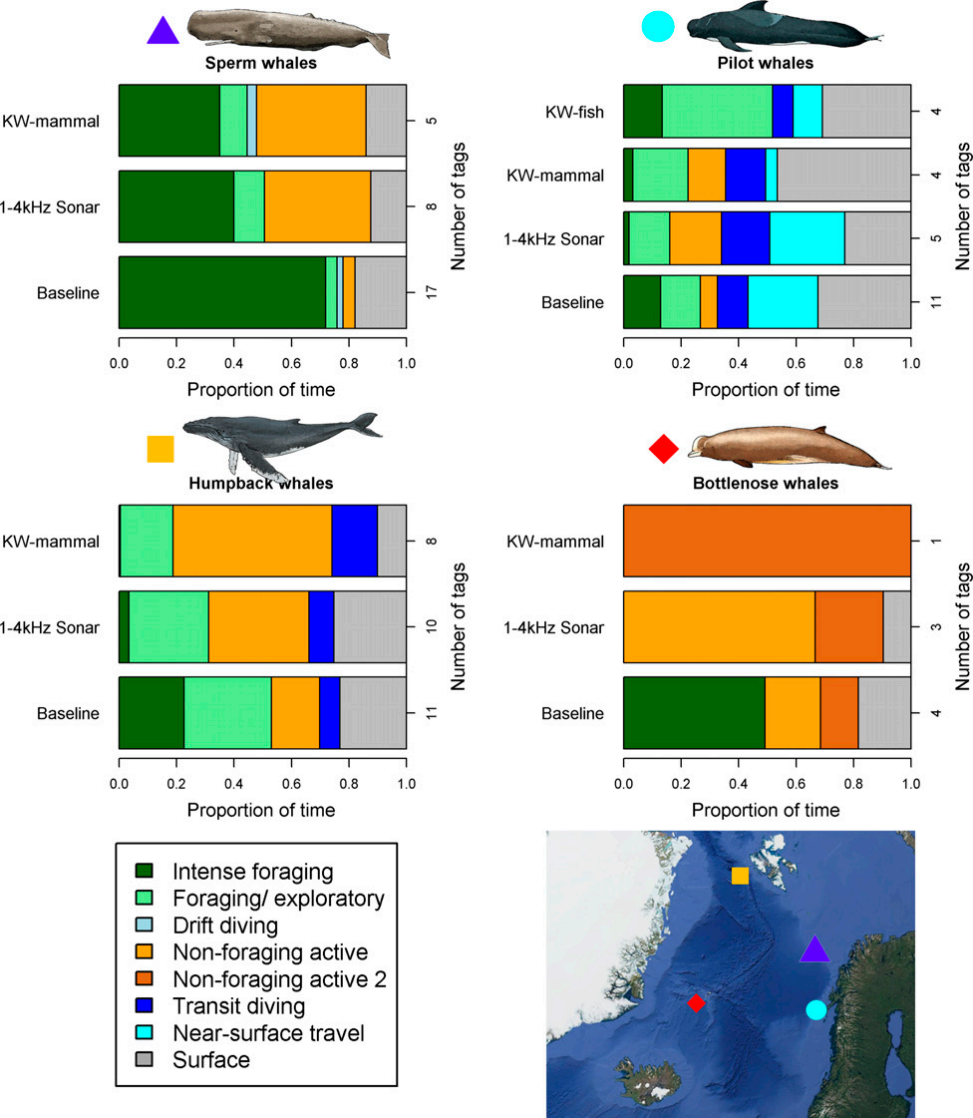
Time budgets of four cetacean species tagged in the North Atlantic. Each panel shows the mean proportion of time in different behavioral states during dives in baseline, sonar, and killer whale playback periods. Numbers to the *Right* within each panel indicate the number of tag records used. Note that intense foraging (dark green) time during KW-mammal playbacks and 1- to 4-kH sonar were consistently lower than during baseline for all species, but it was not lower during KW-fish playbacks to long-finned pilot whales. The *Bottom Right* panel indicates the study area for each species.

For individuals of all four species, time spent in intense foraging was markedly lower during exposure to both stimuli than pre-exposure baseline periods (Fig. 2). Treating individuals of all species together, the odds of intense foraging was lower by a similar amount during both 1- to 4-kHz sonar (−79.5%, Wald = 12.2, *P* < 0.001, *n* = 26) and KW-mammal (−81.7%, Wald = 7.5, *P* = 0.01, *n* = 18) treatments. In contrast, there was no decline in intense foraging during no-sonar (Wald = 3.9, *P* > 0.05, *n* = 25) and broadband noise (Wald = 0.18; *P* > 0.05, *n* = 13) controls. Playback of nonpredatory, fish-eating killer whale sounds (KW-fish) to pilot whales also did not lead to a reduction in intense foraging (Fig. 2; see also reference 25). This demonstrates there were clear overall reduced feeding responses specifically elicited by sonar and KW-mammal.

**Fig. 3.**
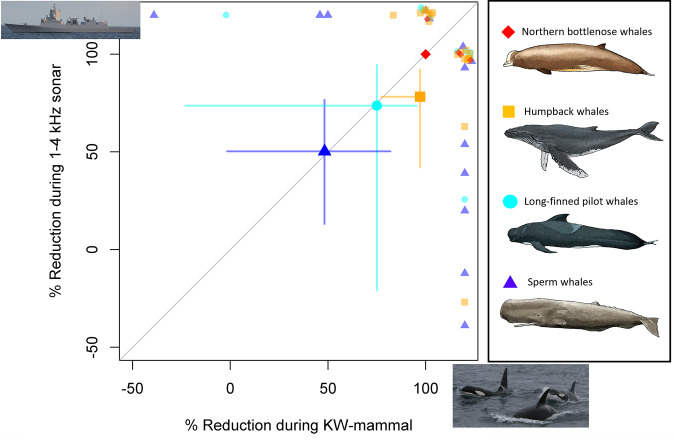
Species-average reductions (95% CI error bars) in intense-foraging dive time during 1- to 4-kHz sonar exposures (*y* axis) related to species-average reductions during playback of predatory killer whale sounds (*x* axis). The gray line is 1:1. Observed changes during single-exposure sessions (note a few sessions had increased intense-foraging) are indicated by symbols *Above* (for KW-mammal playbacks) and to the *Right* (for 1- to 4-kHz sonar) in the figure, with jitter added to the data points at 100% reduction to aid the visibility of those data points.

During exposure, time in intense foraging dives was lower across whale species, with highly concordant species-average reductions to sonar and KW-mammal treatments falling near a 1:1 line (Fig. 3 and *SI Appendix*, Fig. S1). Bottlenose whales had the strongest responses with a 100% loss of intense foraging time during exposure to both stimuli, followed by humpback whales and long-finned pilot whales. Sperm whales had the lowest responses to both stimuli, reducing intense foraging by ∼50% relative to baseline levels. The correlation across species (*r* = 0.92; *n* = 4) supports our prediction that ecoevolutionary contexts that predispose species responsiveness to predator sounds also shape their responsiveness to anthropogenic signals.

Consistent with the species-level correlation, quasilikelihood under the independence model criterion model selection showed that species as a factor covariate and species-average responses to KW-mammal as a continuous covariate were similarly strong predictors of individual responses to 1- to 4-kHz sonar (*n* = 26; *SI Appendix*, Table S4). This result indicates that individual responses to sonar (with the observed variability within species) could be predicted by the species-level responsiveness to killer whale sounds. Thus, the species-level effect was supported when accounting for variations in time spent foraging during baseline and sonar exposures across different tag deployments.

## Discussion

The results of this multispecies quantitative analysis show that knowledge of how a species or population responds to predator sounds can be used to predict responses of individual whales to anthropogenic noise. This implies not only empirical support for the risk disturbance hypothesis but also its application to identify at-risk species to noise pollution. Measuring reduction in intense feeding activities during playbacks of predatory killer whale sounds provided a direct assay of perceived immediate predation risk for each species (35), as the direct observation of at-sea predation pressure in these species is not currently feasible. The strong correlation across species in the average reduction in intense feeding during presentation of 1- to 4-kHz sonar and predatory killer whale sounds was surprisingly close to a 1:1 line. The average values provide the best point estimate of species-level responsiveness but ignore variability within each study population. This variability was considered in the generalized estimating equation (GEE) models, which indicated that individual responses to 1- to 4-kHz sonar could be statistically explained by the species average responsiveness to killer whale playbacks. The high observed variability in responsiveness to sonar is expected from a theoretical standpoint, as the fitness pay-offs of choosing life over dinner depend both on the individual (e.g., sex–age and body condition) and environmental context (e.g., food availability), as well as predation risk. Individuals of the most sensitive species (northern bottlenose whale) had no variation in their responses, indicating that life dominated in the presence of an acoustic threat, while less-responsive species had a wide within-species variation in changes in intense feeding—dinner—during exposures (Fig. 3). Highly concordant species-average reductions in intense feeding during exposure to predatory killer whale sounds and 1- to 4-kHz naval sonar imply that these mesopredators perceive the level of threat from sonar to be similar to predation risk, warranting an equivalent trade-off between food and safety.

As this study focused on 1- to 4-kHz sonar signals, we might expect humpback whales to have the best hearing in the frequency band of the sonar (36) and hence be more sensitive to disturbance by those sounds. Instead, the most sensitive species to both stimuli in our study was the northern bottlenose whale. An earlier datapoint consistent with our finding was reported for a Blainville’s beaked whale (15) with cessation of feeding during both experimental sonar exposure and subsequent playback of killer whale sounds. Beaked whales are known to be particularly sensitive to disturbance from naval sonar (12, 16), with a documented link between naval sonar exercises and strandings (37) possibly due to behavioral responses leading to decompression sickness (38). Such costly responses to anthropogenic sounds can be understood within the risk disturbance hypothesis framework as the sonar triggering antipredator responses that would have been adaptive in the face of real predation risks (39, 40). Unlike the other species in our study, beaked whales likely have limited fight defense mechanisms against killer whale predators; instead, extreme avoidance responses reduce predation risk (31). Such strong antipredator responses are also seen in smaller baleen whale species (30), which are also very responsive to naval sonar exposure (41). Humpbacks, particularly calves, are regular prey of mammal-eating killer whales (42), consistent with their position as the second-most sensitive species in our study. We suggest that the least-reactive species in our study, namely, sperm and long-finned pilot whales, tolerated more risk in our study as they have effective fight responses due to their large body size and group sizes, respectively.

Cetaceans evolved within underwater soundscapes rich in public acoustic information, but with limited physical refugia (i.e., diving to depth to reduce visual detection (43, 44), and thus had a strong selective advantage to recognize acoustic cues of their predators and prey (43). Cetacea evolved sensitive underwater hearing over a wide frequency spectrum (reviewed in reference 9). The harbor porpoise, for example, can hear sounds as low as 1 kHz, despite producing narrow-band high-frequency echolocation clicks at 125 kHz (45). Both of those adaptations are predictable evolutionary consequences of an underwater soundscape of fear; narrow-band high-frequency echolocation clicks reduce their detectability by predatory killer whales, and low to midfrequency hearing enables their detection of killer whale sounds. Antipredator responses, such as fleeing and cessation of feeding, carry energetic and missed opportunity costs, and yet they evolved because they reduce mortality risk from predators, thereby optimizing fitness (28; Box 1). These adaptive responses are now being triggered by generalized threatening stimuli contained within anthropogenic sources (6).

Box 1. The risk-disturbance hypothesis explains how antipredator adaptations to an underwater soundscape of fear predispose species to behavioral disturbance from anthropogenic noise. 
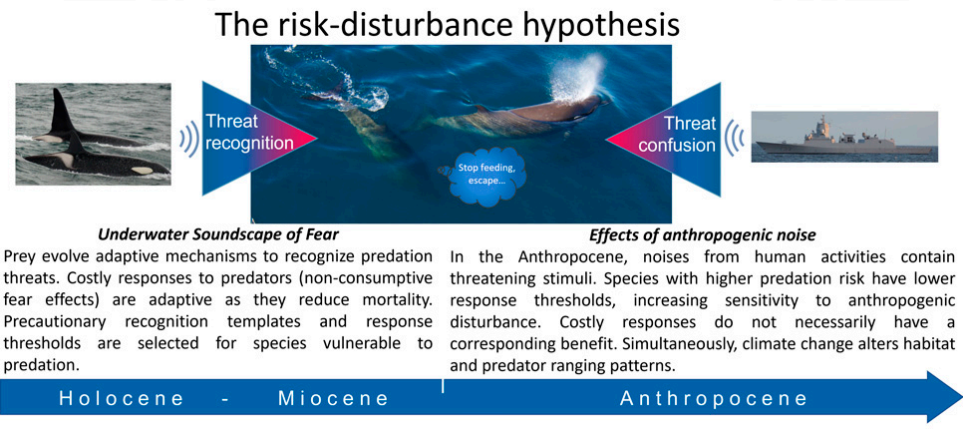

Our experimental support for the risk disturbance hypothesis indicates that factors that alter mesopredator cetaceans’ aversion to predation risk will also influence their responsiveness to anthropogenic disturbance. Foraging time budgets of individuals during sonar exposures were variable but partly predicted by their species-average response to playbacks of predator sounds. If the results were driven by variation in antipredator adaptations, we predict that cetacean species that rely upon crypsis and escape antipredator behaviors and species with high background predation risk will be most sensitive to disturbance by anthropogenic noise. This includes all narrow-band high-frequency echolocating odontocetes, such as the harbor porpoise (46), Monodontidae (belugas and narwhals), the minke and sei whales, and beaked whale species, several of which have been shown to strongly respond to noise (12, 17, 18, 41). Although our study focused on interspecific differences, we can expect that the socioecological context of exposure within species will also drive variation in how cetaceans respond to sonar (4, 12). The presence of vulnerable calves in humpback whale groups (42), for example, can affect how they respond to both sonar (47) and predatory killer whale sounds (24). A greater understanding of contexts that influence predation risk and its tolerance in mesopredator cetaceans, such as proximity to refugia when available (48) or individual body condition (49), should improve our ability to predict sensitivity to noise disturbance, particularly responses that can occur at low received sound levels (50).

In our study, matched responses to 1- to 4-kHz sonar and predatory killer whale sound playbacks indicate that the mesopredator cetaceans have not adjusted their threat response by learning when novel anthropogenic threats do not pose true predation risks (51). The initial sensitivity is expected to wane as individuals habituate to or learn to tolerate anthropogenic sounds with experience (7, 52), although the timescales for this are not well understood and could vary with predation risk itself (53). The novel use of anthropogenic sources should have the most extreme impacts within pristine environments (16, 54). This is a particular concern for Arctic cetaceans, with both killer whales (55) and humans (3) increasingly able to access Arctic waters due to melting sea ice. There is limited knowledge on how free-ranging Arctic seals, which are also potential prey for killer whales, respond to anthropogenic disturbance, and yet many pinniped species are becoming increasingly vulnerable due to climate change (56). Several Arctic mesopredators use crypsis and flight as primary mechanisms to avoid predation by killer whales (48, 55, 57), and similar responses have been reported to icebreaker noise (58) and airguns used in oil and gas exploration (59, 60). We extrapolate that these Arctic specialists will face a looming double-whammy impact of increased direct predation and potentially severe maladaptive responses (39, 61) to novel anthropogenic sounds.

## Materials and Methods

### Field Data Collection.

All field procedures were permitted by the Norwegian Animal Research Authority and approved by the University of St Andrews Animal Welfare and Ethics Committee.

Individuals of the four species of whales (sperm whales, *Physeter macrocephalus*; humpback whales, *Megaptera Novaeangliae*; long-finned pilot whales, *Globicephala melas*; and northern bottlenose whales, *Hyperoodon ampullatus*) were studied in multiple field efforts from 2008 to 2017 as part of the 3S collaborative research project (*SI Appendix*, Table S1). Upon sighting, a randomly selected whale was approached for tagging using a small boat. Tags were attached to the whales using four suction cups, via a carbon fiber pole or an arial remote tagging system (ARTS). All tags included a multisensor datalogger (Dtag, version 2 or 3) which recorded sound (96 or 192 kHz, 16 bits) as well as pressure, three-axial acceleration and three-axial magnetic field strength (50 or 250 Hz). Sensor data were decimated to a standard sampling rate of 5 Hz. Tagged animals were tracked from a research vessel to position the source boat for experimental naval sonar transmission or playback of killer whale sounds. Data used for baseline are those collected from immediately after the tag boat was recovered by the main research vessel, which is when tagging effects cease in sperm whales (62), up to the start of the first experimental exposure. Upon detachment, each tag was recovered using a radio transmitter integrated into the tag unit.

Sonar transmissions were in the 1- to 4-kHz band, and only periods when the received sound pressure level exceeded 120 dB re 1µPa were included as 1- to 4-kHz sonar exposure treatments. Most experimental exposures were conducted using the Socrates sonar source towed by the vessel RV HU Sverdrup II and transmitted a 0.5- to 1.0-s duration, 1- to 2-kHz hyperbolic upsweep every 20 s. Sonar exposure sessions lasted 15 to 24 min for bottlenose whales, 10 min for humpback whales, and 30 to 80 min for pilot and sperm whales. During each exposure, the sound source level was gradually increased from 150 dB re 1µPam to a maximum of 214 dB re 1µPam. Sonar transmission started at a planned distance of 8 km from each tagged whale, and the vessel source moved toward the whale at 8 knots (4 m/s). This exposure protocol led to a gradual increase in received levels at the tagged whale. No adjustments to the course of the source vessel were made once it was 1 km from the position of the tagged subject whale. Control no-sonar approaches were made following the same procedures, except that no signals were transmitted from the Socrates sonar system. Due to technical limitations, transmissions made in 2015 and 2016 with bottlenose whales were made from the deck of MV Donna Wood with the vessel drifting. The 2015 exposure did not include a ramp-up period [see experiment 2015-2 in Wensveen et al. (16)]. The 2016 bottlenose whale exposure used a 1.5-s duration tonal signal over 3.4 to 3.9 kHz (see experiment 2016-1 in reference 16).

Killer whale sound playbacks were conducted from a separate dedicated vessel, with a planned position roughly 45 degrees off the whale horizontal trajectory at approx. 800-m distance from the whale at start of playback (24, 25, 27). Sounds were typically transmitted for 15 min using a Lubell underwater speaker transmitting at natural sound levels for killer whales (145 to 155 dB re 1µPam). Monitoring recording of playbacks was simultaneously conducted to ensure that sounds were faithfully broadcasted by the playback system. Two pairs of KW-fish playbacks that were conducted in a short succession (with only 5- and 11-min breaks in between the playbacks) were treated as two continuous playbacks. Killer whale playbacks were prepared from natural recordings of feeding killer whales obtained using Dtags. For KW-mammal, the stimuli were of mammal-feeding killer whales tagged in southeastern Alaska. For KW-fish, the sounds were of herring-feeding killer whales in Vestfjord, Norway. Control broadband noise playbacks were generated using the background noise periods of Dtag recordings, normalized to the same sound pressure source level as for the killer whale sounds playbacks.

### Data Processing.

Following standard procedures, pressure data were converted to depth, and the pitch of the tagged whale was calculated from the three-axis accelerometer data corrected for tag placement on the body of the whale. Fluke strokes were detected using an automated detector based upon cyclic variations in pitch (63), with detection parameters determined manually for each tag record by inspecting the magnitude of the stroke signals within the pitch record. Circular variance of roll and low-pass filtered pitch were calculated within R package CircStats (64). The pitch was low-pass filtered to remove the fluke stroke signal, using fourth-order Butterworth filter with threshold frequency set by inspecting periodograms of the raw pitch values. For the three odontocete species, audio files recorded by Dtags were inspected aurally and visually by expert analysts using spectrograms to identify the start and stop times of foraging sounds produced by each tagged whale, separately from those produced by other whales. Foraging echolocation search clicks and buzzes were ascribed to the tagged whale depending upon the sounds’ relative amplitude and spectral characteristics. For tagged humpback whales, lunges were detected as noise peaks that were followed by at least a 12-dB drop within 5 s (33).

For long-finned pilot whales and humpback whales, dives and associated breaths were detected following reference 65. For both species, horizontal movement was summarized for each interbreath interval as the turning angle between tag-recorded headings at consecutive breath times (over a 0.5-s averaging window) and mean horizontal speed of the focal group between visually recorded locations. In humpback whales, a moving average of three 0.5-s analysis windows was used to smooth horizontal speed before summarizing it for each interval. Humpback whale swim speed was estimated from flow noise following reference 47.

Sonar received levels were measured from recordings on the Dtags, following the methods detailed in reference 47. Sonar exposure sessions were considered to start once SPLmax reached 120 dB re 1µPa, and the sonar exposure period until that point was not included in the analysis.

### Dive State Classification.

To estimate time spent in intense foraging for each tagged whale, time series data were classified into activity states using tailored algorithms for each species. Sperm whale data were classified at 1-min time resolution into six behavior states, including surfacing, by fitting a hidden Markov model (HMM) in a Bayesian framework (62). For all other species, threshold-based dive detection was carried out before fitting a dive-by-dive HMM in a maximum likelihood framework following reference 65.

The sperm whale state-based model allowed decoding the time series into the following six states: surface, descent, layer-restricted search (LRS), ascent, resting/drifting, and nonforaging active state. The model included a state-dependent random walk for depth, state-dependent probability of clicking (buzz and regular clicks treated the same), and state-dependent relationship between absolute pitch and vertical speed. To represent vertical transit, positive and negative drift parameters were estimated during descent and ascent, respectively. A lack of relationship between pitch and vertical transit was specified for surface and resting states. A single Markov transition probability matrix was assumed, except for increasing the probability of surfacing with decreasing depth. Please see reference 62 for further details of the state-based model structure and fitting. Intense foraging for sperm whales was defined as time spent in LRS and any time spent in descent or ascent when adjacent to LRS. Other descent and ascent behaviors were considered exploratory, less intense foraging (*SI Appendix*, Fig. S2 and Table S2).

Long-finned pilot whale dives were defined as submergences deeper than 5.3 m or longer than 37.8 s in duration. The diving thresholds were derived by classifying and characterizing dives vs. near-surface movements (breathing behavior) using two-state mixture model (65). Dive types were then estimated in a multivariate HMM. The HMM specified state-dependent likelihoods for dive summary metrics selected to reﬂect the animals’ diving effort (dive depth, duration, pitch variance), horizontal swimming effort (horizontal speed, turning angle), foraging behavior (presence/absence of echolocation), and social behavior (group size, presence/absence of social sounds, and presence/absence of tight spacing within the group). The lowest Bayesian information criterion (BIC) model included no covariates or random effects and four dive types. Intense foraging for pilot whales was defined as the dive type with the highest probability of echolocation clicks and deepest depth distribution (mean 300 m). Exploratory dives had the second highest probability of clicking but were considerably shallower (<40 m; *SI Appendix*, Fig. S1 and Table S2).

Humpback whale dives were defined as submergences deeper than 4.9 m or longer than 34.6 s in duration. As in reference 65, the diving thresholds were calculated as 98% of depth and duration of near-surface movements. Dives vs. near-surface movements were classified in a two-state mixture model (65), wherein interbreath interval was specified a Weibull distribution, vertical displacement an exponential distribution, and circular variance of roll a beta distribution. To ensure the algorithm did not terminate at a local minimum, the model was fitted 100 times with different initial values, and the stability of the resulting likelihoods was monitored visually.

The dive-type HMM for humpback whales included the following dive summary metrics and distributional assumptions: dive duration (Weibull), dive depth (gamma), fluke stroke rate (gamma), mean horizontal speed (gamma), median vertical speed (gamma), mean swim speed (gamma), circular variance in pitch (beta), change in heading (Von Mises), and presence/absence of lunging (Bernoulli). As with the mixture model, the HMMs were fitted 100 times with different initial values. Models with up to five states were fitted, with AIC and BIC supporting the maximum number of states. Inspection of the model outputs revealed that those dives classified as one of two types of foraging dives (with lunging probability of >0) in the four-state model were all classified as one of three types of foraging dives in the five-state model. The four-state model foraging categories obtained slightly greater lunging probabilities (0.9 and 0.57) than the five-state model (0.89, 0.6, and 0.54). Therefore, the four-state model state with the highest lunging probability was selected to infer time spent in intense foraging state (*SI Appendix*, Fig. S1 and Table S2).

Northern bottlenose whale dives were defined as submergences exceeding 10 m in depth (roughly one body length). The dive-type HMM for bottlenose whales included the following dive summary metrics: dive depth (Weibull), vertical speed (Weibull), circular variance in roll (Beta), presence/absence of a buzz (Bernoulli), presence/absence of a jerk peak (Bernoulli), and presence/absence of clicking (Bernoulli). Models with up to 4 states were fitted (again, 100 times with different initial values), with BIC supporting 3 states. In this model, only a single foraging dive type with probability of buzzing of >0 was supported, which was then used to calculate time spent in intense foraging for this species (*SI Appendix*, Fig. S1 and Table S2).

### Statistical Methods.

The aim of the statistical modeling was to 1) quantify any species differences in cessation of foraging during the experimental exposures relative to baseline, i.e., the magnitude of foraging time trade-offs and the consistency of such trade-offs across different individuals; and 2) test whether the response intensity to playbacks of predatory killer whale sounds explained cessation of foraging response to 1- to 4-kHz sonar across the four species, accounting for variability across individuals within each species. For each species, the scaled response intensity covariate (hereafter, KW-RI) was calculated as the difference between average time spent foraging during killer whale sound playbacks and the average pre-exposure baseline foraging time budget, divided by the average foraging time during baseline periods. The same calculation was used to visualize model predictions for each species (difference in estimated time spent foraging during exposure vs. preexposure baseline, scaled by the average baseline value in Fig. 2). Deployment-specific response intensity to sonar (Fig. 3) and other exposures (*SI Appendix*, Fig. S1) were calculated as the change in foraging time during exposure compared to pre-exposure baseline, again scaled (divided) by the average baseline value for each species.

The proportion of time spent in the intense foraging behavior state was modeled as a binomial response variable. The analysis units were consecutive 1-h time periods during the baseline and the exposure session. The time bins were extracted so that the final pre-exposure time bin would match the end of the baseline period. Any residual time bin (<1 h in duration) at the beginning of each baseline period was included if its duration was 10 min or longer.

The duration of each baseline period (1 h) and sound exposure (0.2 to 1.3 h) was included as weights in the model. Repeat exposures of the same sound exposure type (1- to 4-kHz sonar, no-sonar, KW-mammal, KW-fish, and broadband noise playback) were excluded from the model fit.

The models were fitted in a GEE framework, which allows the estimation of individual-average parameters with standard errors that account for correlation within individuals. The models were specified with the tagged individual as a panel variable. The robust Sandwich estimator was used to extract SEs, and independence was specified as the working correlation. Parametric bootstrap with 1,000 iterations was used to generate 95% confidence intervals for model predictions.

First, a model was fitted to test the effect of the different sound and control exposures on time spent in intense foraging state for each species. This model was fitted including baseline, 1- to 4-kHz sonar exposures (coded here as pulsed active sonar [PAS]), no-sonar control approaches, broad-band noise playbacks (BBN), and KW-mammal playbacks from all the four species. Exposures were considered “on” after received sound pressure level (SPL) of 120 dB re1µPa was reached. The candidate main effects included species (a factor covariate), presence/absence of no-sonar (NS) exposure, presence/absence of PAS exposure, presence/absence of control playbacks (PB_BBN), and presence/absence of KW-mammal (PB_KWM). Species was included as an interaction to test species differences in response intensity to each exposure type.

The statistical model to test effects of each exposure type on time spent foraging is as follows:

Foraging1∼Species+NS+PAS+PB_BBN+PB_KWM+Species2:NS+Species:PAS+Species2:PB_BBN+Species:PB_KWM

A second set of models was fitted to test whether species responsiveness to playbacks of killer whale sounds predicted responsiveness to sonar, given an observed variation in response intensity across individuals within each species. These models excluded data during control exposures (which were tested in the first model) and playbacks of killer whale sounds, which were instead used to calculate the covariate of interest KW-RI. KW-RI represented species-average response intensity to killer whale playback. The following three model structures were considered:

$$\begin{array}{l} \text{Foraging1}∼\text{Species}+\text{PAS}\\ \text{Foraging1}∼\text{Species}+\text{KW}-\text{RI}:\text{PAS}\\ \text{Foraging1}∼\text{Species}+\text{Species}:\text{PAS} \end{array}$$

The first model represents the hypothesis that each species has a specific baseline level of foraging but with an equivalent change in foraging time during PAS. The second model tested the hypothesis that the change in foraging time during PAS was a function of the species response to killer whale playbacks (KW-RI:PAS interaction). The third model tested a species-specific response to sonar (Species:PAS interaction). We did not consider a main effect for KW-RI (i.e., whether playback response could explain baseline level of foraging).

## Supplementary Material

Supplementary File

## Data Availability

The datasets generated during and analyzed during the current study, and code used for analysis, are available on Zenodo and GitHub at the following link: https://doi.org/10.5281/zenodo.5996290. All other data are included in the manuscript and/or *SI Appendix*.
