# Supplementary material for: Behavioral responses to predatory sounds predict sensitivity of cetaceans to anthropogenic noise within a soundscape of fear
Source: Proc Natl Acad Sci U S A. 2022 Mar 21;119(13):e2114932119. doi: 10.1073/pnas.2114932119 (PMC9060435; doi:10.1073/pnas.2114932119)
Supplement: Supplementary File [file pnas.2114932119.sapp.pdf]

PNAS
www.pnas.org

Supplementary Information for

Behavioral responses to predatory sounds predict sensitivity of cetaceans to anthropogenic noise within a soundscape of fear

Authors: Patrick J.O. Miller[1*], Saana Isojunno[1†], Eilidh Siegal[1], Frans-Peter A. Lam[2], Petter H. Kvadsheim[3], Charlotte Curé[4]

Affiliations:

[1]Sea Mammal Research Unit, U of St Andrews, St Andrews Fife KY16 9QQ, UK.

[2]Acoustics & Sonar, TNO, The Netherlands.

[3]Norwegian Defence Research Establishment, Norway.

[4]UMRAE, Cerema-University Gustave Eiffel, Strasbourg, France.

*Correspondence to: pm29@st-andrews.ac.uk

†Isojunno and Miller are joint first authors.

This PDF file includes:

Figures S1 and S2
Tables S1 to S4

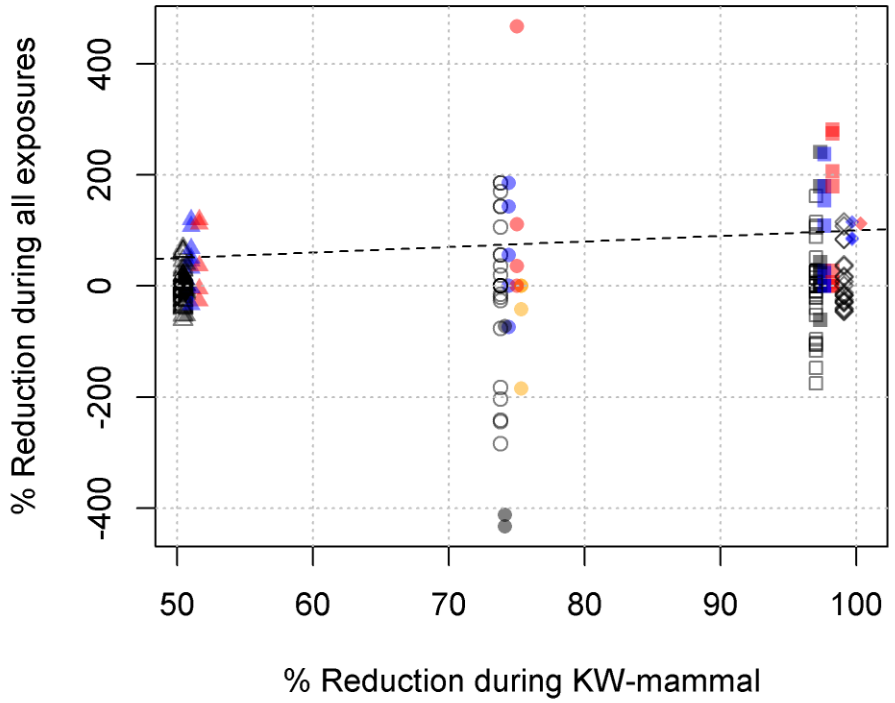

Fig. S1. Percentage reduction in time spent intense-foraging (scaled response intensity: RI) of 1h baseline periods and exposures (y-axis), as a function within-species average RS of playbacks mammal-eating killer whale (KW-mammal) vocalizations. The x-axis values for each exposure type (controls and sound exposures) are offset by a small constant to enable visualization of all data points. Symbols indicate the four species (diamonds: bottlenose whales, squares: humpback whales, circles: pilot whales, triangles: sperm whales). Hollow symbols indicate baseline periods, while solid symbols indicate experimental sessions **Black**: no-sonar, **Blue**: 1-4 kHz sonar, **Red**: KW-mammal, **Orange**: KW-fish). The black dashed line shows the one-to-one line of equivalence between the two axes (expected if perceived risk from sonar = perceived risk from KW playbacks). Note the key pattern that red datapoints are centered around the 1:1 line for each species, while control datapoints are centered around zero reduction.

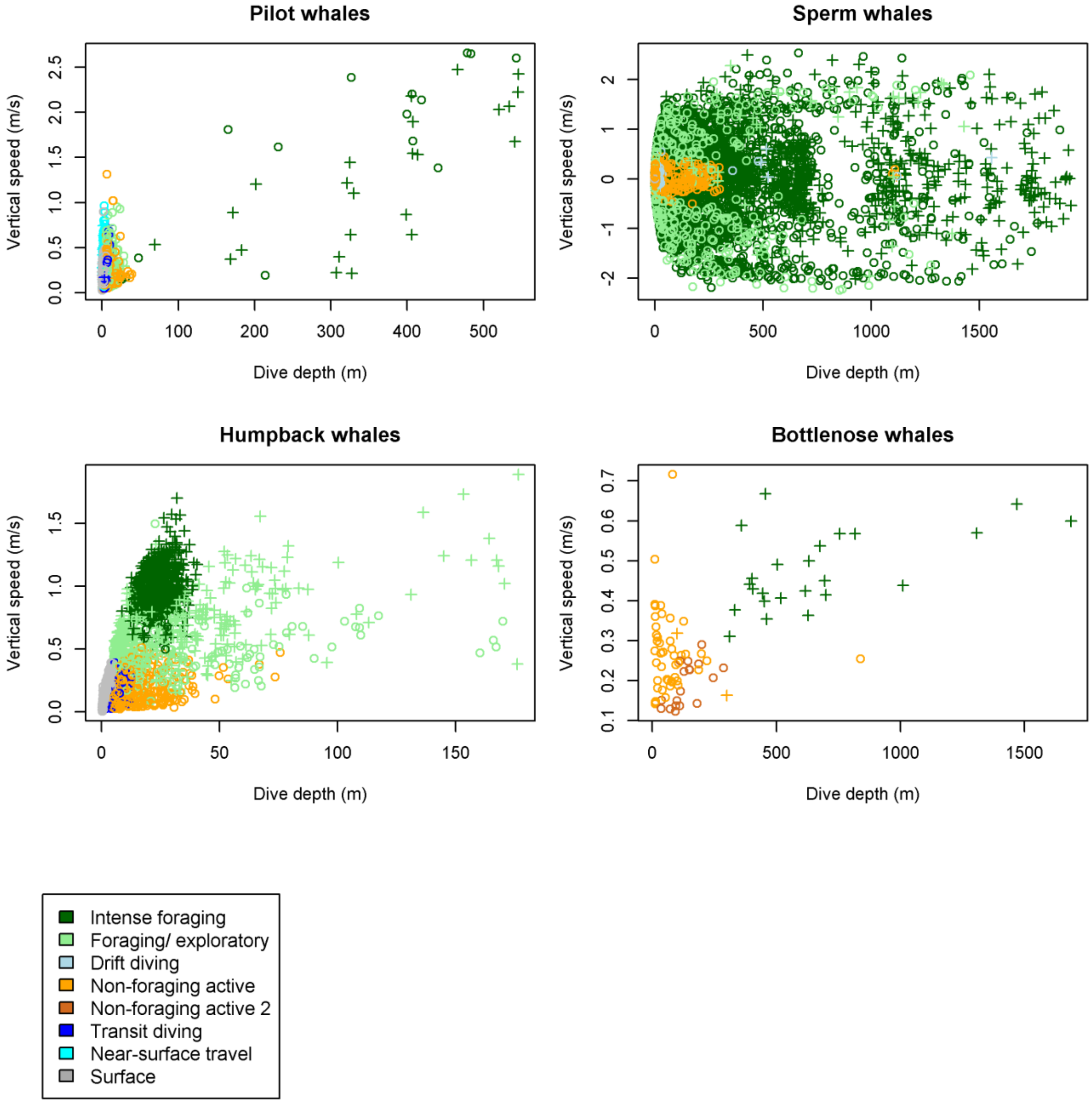

Fig. S2. Data parameters for dive states for the 4 different species.

Dive depth (m) and median vertical speed (m/s) of analyzed behavior states for each species (full dive types are shown for all species except sperm whales, for which 1-min time bins within dives are shown). Crosses show dive times/time bins with feeding (buzzes for all species except humpback whales, for which lunges are shown).

Table S1. Summary of Dataset

Table 1. Summary of data. Depl. refers to deployments, NS to no-sonar, BBN to broadband noise, ctrl to controls, KWM to KW-Mammal experiments and KWF to KW-Fish experiments. Species are northern bottlenose whale (NBW), humpback whale (HW), long-finned pilot whale (LPW), and sperm whale (SW).

				# of exposures					Session duration (min)		
Species	Year	# depl.	Baseline dur (h)	NS ctrl	1-4 kHz sonar	BBN ctrl	KWM	KWF	1-4 kHz sonar	KWM	KWF
NBW	2013	1	7.7	0	1	0	0	0	24	NA	NA
	2015	2	5.7	0	1	0	1	0	15	15	NA
	2016	1	3.3	0	1	0	0	0	NA	NA	NA
HW	2011	3	17.2	3	3	3	3	0	9	15	NA
	2012	8	30.6	7	7	5	5	0	9	15	NA
LPW	2008	3	5.2	1	3	0	0	1	46	NA	48
	2009	2	8.1	2	2	0	0	1	30	NA	37
	2010	2	4.4	0	0	2	0	2	NA	NA	14
	2013	3	6.2	0	0	1	3	0	NA	14	NA
	2014	1	2.3	0	0	0	1	0	NA	15	NA
SW	2008	1	1.8	0	1	0	0	0	59	NA	NA
	2009	3	9.0	2	3	0	3	0	41	20	NA
	2010	2	18.9	0	0	2	2	0	NA	15	NA
	2016	4	20.2	4	1	0	0	0	32	NA	NA
	2017	7	38.8	6	3	0	0	0	32	NA	NA

Table S2. Definition of foraging states

Species	Intense foraging	Foraging/exploratory
Long-finned pilot whales	Dive-by-dive HMM "Foraging" dive category (deepest, with highest probability of clicking, and most active)	HMM "Exploratory", similar to foraging but shallower
Humpback whales	Dive-by-dive HMM "Foraging 1" dive, with the highest probability of lunging	HMM "Foraging 2" dive, active but not as many lunges (50-50 chance)
Sperm whales	Minute-by-minute HMM classification of layer-restricted search (LRS), and any descent/ascent state adjacent to LRS state	HMM-classified descent and ascent behaviour that wasn't adjacent to LRS
Bottlenose whales	Dive-by-dive HMM "Foraging" dive category (deepest, with highest probability of buzzes and jerks).	None detected by HMM.

Table S3. GEE-GLM statistical model output (all data)

Model	Parameter	Estimate	SE	Wald statistic	p-value
A	Intercept (Species - SW)	0.86	0.20	18.95	<0.001
	Species - LPW	-2.54	0.37	46.15	<0.001
	Species - HW	-2.48	0.51	23.38	<0.001
	Species - BW	-1.03	0.24	18.48	<0.001
	NS	0.56	0.28	3.90	0.05
	PAS	-1.59	0.45	12.17	<0.001
	PB_BBN	-0.26	0.60	0.18	0.67
	PB_KWM	-1.70	0.62	7.48	0.01
B	Intercept (Species - SW, Species2 - SW)	0.86	0.20	18.13	<0.001
	Species - LPW	-2.66	0.44	35.93	<0.001
	Species - HW	-2.45	0.53	21.49	<0.001
	Species - BW	-1.00	0.25	16.30	<0.001
	NS	0.36	0.26	1.94	0.16
	PAS	-1.48	0.55	7.32	0.01
	PB_BBN	-0.28	1.08	0.07	0.80
	PB_KWM	-1.42	0.77	3.45	0.06
	NS:Species2 - LPW	1.70	0.68	6.35	0.01
	NS:Species2 - HW/BW	-0.99	0.64	2.37	0.12
	Species - LPW:PAS	0.04	1.05	0.00	0.97
	Species - HW:PAS	-0.19	0.76	0.06	0.80
	Species - BW:PAS	-9.89	0.57	305.31	<0.001
	PB_BBN:Species2 - LPW	-9.44	1.15	67.72	<0.001
	PB_BBN:Species2 - HW	0.35	1.34	0.07	0.79
	Species - PW:PB_KWM	-0.08	1.19	0.00	0.95
	Species - HW:PB_KWM	-2.32	1.38	2.84	0.09
	Species - BW:PB_KWM	-9.95	0.78	163.20	<0.001

Model A: Foraging ~ Species + NS + PAS + PB_BBN + PB_KWM, Model B: Foraging ~ Species + NS + PAS + PB_BBN + PB_KWM + Species2:NS + Species:PAS + Species2:PB_BBN + Species:PB_KWM, where NS = no-sonar, PAS = presence-absence of sonar exposure, PB = playback, BBN = broadband noise control, KWM = mammal-feeding killer whale playback, SW = sperm whale, BW = bottlenose whale, HW = humpback whale, LPW = long-finned pilot whale. The northern bottlenose whale dataset did not include NS or BBN controls, therefore it was not included in those interactions.

Table S4. GEE-GLM statistical model output (baseline & sonar)

Model	QIC/ QICu	Term	Df	X^2	P(>|Chi|)
1	257/ 247	Species	3	53.9	<0.001
		PAS	1	11.9	<0.001
2	256/ 246	Species	3	53.9	<0.001
		KW-RI:PAS	1	11.0	<0.001
3	257/ 252	Species	3	54.0	<0.001
		Species:PAS	4	6395	<0.001

KW-RI: species response intensity to playback of killer whale sounds, calculated as the difference between average baseline and average playback intense-foraging time, divided by the average baseline intense-foraging time. KW-RI:PAS is the interaction between KW-RI and pulsed active sonar PAS (presence/absence of sonar exposure), representing the dose of the sonar exposure as a function of response intensity to playback of killer whale sounds. Similarly, Species:PAS is the interaction between Species and pulsed active sonar, estimating a species-specific dose of sonar exposures. Note the second model has the strongest support using either QIC or QICu values.
